# TORCH-R trial protocol: hypofractionated radiotherapy combined with chemotherapy and toripalimab for locally recurrent rectal cancer: a prospective, single-arm, two-cohort, phase II trial

**DOI:** 10.3389/fonc.2023.1304767

**Published:** 2023-11-20

**Authors:** Juefeng Wan, Ruiyan Wu, Miaomiao Fu, Lijun Shen, Hui Zhang, Yan Wang, Yaqi Wang, Shujuan Zhou, Yajie Chen, Fan Xia, Zhen Zhang

**Affiliations:** ^1^Department of Radiation Oncology, Fudan University Shanghai Cancer Center, Shanghai, China; ^2^Department of Oncology, Shanghai Medical College, Fudan University, Shanghai, China; ^3^Shanghai Clinical Research Center for Radiation Oncology, Shanghai Key Laboratory of Radiation Oncology, Shanghai, China

**Keywords:** locally recurrent rectal cancer, immunotherapy, hypofractionated radiotherapy, chemotherapy, SAbR

## Abstract

For patients with locally recurrent rectal cancer (LRRC), the response rate to chemoradiotherapy is 40%–50%. Additionally, only approximately 40%–50% of patients with recurrent rectal cancer are able to undergo R0 resection. Recent studies in locally advanced rectal cancer (LARC) have shown promising synergistic effects when combining immunotherapy (PD-1/PD-L1 antibodies) with neoadjuvant chemoradiotherapy (nCRT). Therefore, incorporating immunotherapy into the treatment regimen for LRRC patients has the potential to further improve response rates and prognosis. To investigate this, the TORCH-R trial was conducted. This prospective, single-arm, two-cohort, phase II trial focuses on the use of hypofractionated radiotherapy, chemotherapy, and immunotherapy in LRRC patients without or with oligometastases. The trial will include two cohorts: cohort A consists of rectal cancer patients who are treatment-naive for local recurrence, and cohort B includes patients with progressive disease after first-line chemotherapy. Cohort A and cohort B patients will receive 25–40 Gy/5 Fx irradiation or 15–30 Gy/5 Fx reirradiation for pelvic recurrence, respectively. Subsequently, they will undergo 18 weeks of chemotherapy, toripalimab, and stereotactic ablative radiotherapy (SABR) for all metastatic lesions between chemoimmunotherapy cycles. Decisions regarding follow-up of complete response (CR), radical surgery, sustained treatment of non-resection, or exiting the trial are made by a multidisciplinary team (MDT). The primary endpoint of this study is the local objective response rate (ORR). The secondary endpoints include the extrapelvic response rate, duration of response, local recurrence R0 resection rate, progression-free survival (PFS), overall survival (OS), and safety and tolerability. Notably, this trial represents the first clinical exploration of inducing hypofractionated radiotherapy, chemotherapy, and immunotherapy in LRRC patients.

**Clinical trial registration:**

https://clinicaltrials.gov/study/NCT05628038, identifier NCT05628038.

## Introduction

The introduction of total mesorectal excision (TME) and the advancement of adjuvant and neoadjuvant treatments have greatly reduced the occurrence of locoregional recurrence in rectal cancer. However, even with these improvements, a small percentage of patients (4%–10%) still experience pelvic recurrence, with or without extrapelvic metastases, particularly in cases where standard initial treatment was not received (1–3). Pelvic recurrence can lead to substantial morbidity, causing severe local symptoms such as pelvic pain, bleeding, and obstruction (4, 5).

The complete resection of a recurrent tumor, known as R0 resection, is the most important prognostic factor that significantly influences survival (6, 7). Achieving R0 resection is crucial, as it leads to 5-year survival rates ranging from 43% to 57%. On the other hand, patients who undergo non-curative resections or receive only palliative treatments do not survive beyond 5 years (8, 9). However, achieving R0 resection can be challenging due to the destruction of anatomical structures in the pelvic cavity and pelvic wall, as well as the invasive nature of recurrent tumors into adjacent tissues. As a result, only approximately 40%–50% of patients with locally recurrent rectal cancer (LRRC) are suitable candidates for surgery with curative intent, and the surgical procedure itself can be quite complex (10, 11).

To improve the rate of R0 resections in patients with LRRC, preoperative chemo(re)radiotherapy is recommended (12). In this treatment approach, radiation therapy is delivered with a cumulative dose of 45–50 Gy in 25 fractions of 1.8–2 Gy [known as long-course radiotherapy (LCRT)] or reirradiation using hyperfractionated doses of 30–39 Gy (1.2–1.5 Gy twice daily with a minimum 6-h interval) (13). In a phase II trial conducted at our center, we reported an overall local response rate of 46.5% and cumulative overall survival rates of 80.1% and 36.5% at 1 and 3 years, respectively, after concurrent chemoradiation in 71 patients diagnosed with recurrent rectal cancer who had not previously received pelvic irradiation (14). Another study revealed an objective response rate of 40% after chemoradiation in patients with recurrent rectal cancer who had not undergone prior chemoradiotherapy (15). For recurrent rectal cancer patients who had previously received pelvic irradiation, the overall local response rate after hyperfractionated radiotherapy was reported to be approximately 40%–50% in various studies (16, 17). Despite achieving good local responses through radiation or reirradiation in recurrent rectal cancer, long-term local control and survival outcomes remain poor.

In recent years, immunotherapy has made significant progress in the treatment of various types of cancers and has emerged as a promising anticancer therapy. Specifically, for patients with microsatellite instability-high (MSI-H) tumors, immunotherapy has shown remarkable efficacy. MSI-H tumors are characterized by a higher tumor mutation burden (TMB) and increased infiltration of tumor-infiltrating lymphocytes (TILs), making them naturally more responsive to immunotherapy (18, 19). However, it is important to note that dMMR/MSI-H tumors account for less than 5% of colorectal tumors, while the majority, over 95%, are classified as microsatellite stable (MSS) tumors. MSS tumors, unfortunately, do not exhibit the same level of sensitivity to immunotherapy alone.

Preclinical studies have provided evidence that radiotherapy can enhance the effectiveness of immunotherapy in cancer treatment (20–25). In recent years, several clinical studies have explored the combination of neoadjuvant chemoradiotherapy (nCRT) with immunotherapy in patients with microsatellite stable (MSS) locally advanced rectal cancer (LARC), and preliminary results have shown promise.

One approach involves combining LCRT, typically delivered at a dose of 50 Gy over 25 fractions, with sensitization using chemotherapy drugs like 5-FU or capecitabine, along with immunotherapy. This combination has demonstrated complete response rates ranging from 30% to 50% in LARC patients (26–32). Studies have also investigated the combination of hypofractionated short-course radiotherapy (SCRT), delivered at a dose of 25 Gy over 5 fractions, with immunotherapy in LARC patients. This approach has shown advantages and achieved a complete response rate of approximately 50% (33–36). Hypofractionated SCRT has been found to have less impact on peripheral blood lymphocytes, thereby promoting the immune system’s antitumor response (37). It inhibits the recruitment of myeloid-derived suppressor cells (MDSCs) into tumors, decreases the expression of PD-L1 on the tumor surface, and achieves a superior tumor growth inhibition rate compared to conventional fractionation (38). Furthermore, the local response observed in these studies serves as an indicator of successful induction of systemic antitumor immunity and the elimination of micrometastases, particularly in the era of immunotherapy (39).

We have designed this study, known as TORCH-R, to evaluate the safety and efficacy of combining hypofractionated (re)irradiation, chemotherapy, and toripalimab in patients with LRRC. In this article, we present the study protocol for TORCH-R, offering a comprehensive description of the trial.

## Methods

### Study design

**Figure 1:**
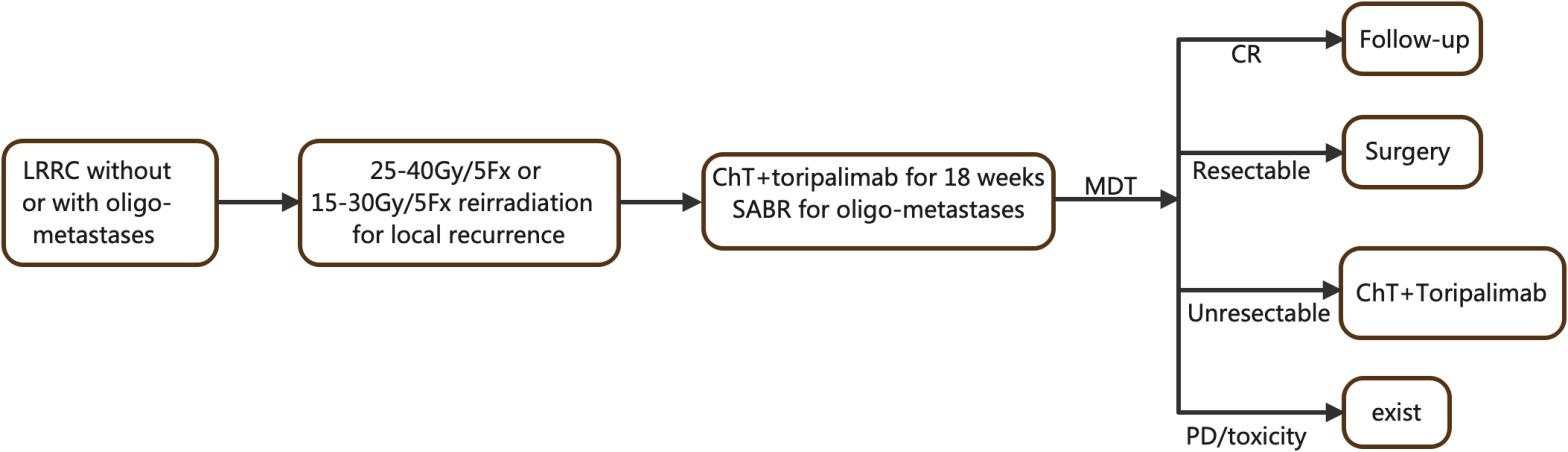
Flowchart of the TORCH-R study.LRRC, locally recurrence rectal cancer; ChT, chemotherapy; SABR, stereotactic ablative radiation; MDT, multidisciplinary team.

Our study is a prospective, single-arm, two-cohort, phase II clinical trial aimed at evaluating the effectiveness of combining (re)irradiation, chemotherapy, and immunotherapy in patients with LRRC, regardless of the presence or absence of oligometastases. For pelvic recurrence, patients will receive either 25–35 Gy/5 Fx irradiation or 15–30 Gy/5 Fx reirradiation if they had previous pelvic radiation. Additionally, patients will undergo 18 weeks of toripalimab and chemotherapy. Stereotactic ablative radiotherapy (SABR) will be administered for all metastatic lesions during the periods between chemoimmunotherapy cycles. A multidisciplinary team (MDT) will then determine the subsequent management plan, which may involve follow-up for complete response (CR), radical surgery, sustained treatment without resection, or exit from the trial. For a visual representation of the study algorithm, please refer to **Figure 1**.

### Patient and public involvement

There will be no involvement of patients or the public in the design, recruitment, assessment, and conduct of this study. The results of the study will be disseminated through peer-reviewed scientific journals and conference presentations, rather than being specifically notified to individual patients.

### Key eligibility criteria

Eligible patients should present histologically or cytologically or MRI/enhanced CT confirmed pelvic recurrence without or with oligometastases. In addition, these patients are required to have an Eastern Cooperative Oncology Group performance status (ECOG PS) of 0 to 1, normal organ function, no history of active autoimmune disease, and no history of immune checkpoint inhibitor (ICI). Please refer to **Table 1** for a comprehensive list of the key inclusion and exclusion criteria.

**Table 1:** Key eligibility criteria for this trial.

Inclusion criteria	Exclusion criteria
**•Aged 18-75** **•ECOG performance status 0–1.** **•Histologically or cytologically or MRI/enhanced CT**-**confirmed pelvic recurrence. According to RECIST 1.1, there is at least one measurable pelvic lesion.** **•Distant metastatic lesions are no more than** five **and no more than** three **metastatic organs.** **•No prior radiotherapy within 6 month**s. **•Previous chemotherapy. Patients Group Cohort A: participants with pelvic recurrence who have not previously been treated with first-line chemotherapy. Cohort B:** p**atients with disease progression or new lesions after first-line chemotherapy.** **•Has an investigator**-**determined life expectancy of at least 24 weeks.** **•Demonstrate adequate organ function (bone marrow, liver, kidney**, **and clotting function) within 7 days before the first administration without using blood products or hematopoietic stimulating factors.** **•Non**-**pregnant or lactating patients. Effective contraceptive methods should be used during the study and within 6 months of the last administration.** **•Fully informed and willing to provide written informed consent for the trial.**	•Neutrophil< 1.5×10^9^/L, PLT< 100×10^9^/L (PLT< 80×10^9^/L in patients with liver metastasis), or Hb< 90 g/L. •TBIL > 1.5 ULN, or TBIL > 2.5 ULN in patients with liver metastasis. •AST or ALT > 2.5 ULN, or ALT and/or AST > 5 ULN in patients with liver metastasis. •Cr > 1.5 ULN, or creatinine clearance< 50 mL/min (calculated according to Cockcroft Gault formula). •APTT > 1.5 ULN, PT > 1.5 ULN (subject to the normal value of the clinical trial research center). •Serious electrolyte abnormalities. •Urinary protein ≥ 2+, or 24-h urine protein ≥1.0 g/24 h. •Uncontrolled hypertension: SBP >140 mmHg or DBP > 90 mmHg. •A history of arterial thrombosis or deep vein thrombosis within 6 months; a history of bleeding or evidence of bleeding tendency within 2 months. •A history of heart disease within 6 months. • Uncontrolled malignant pleural effusion, ascites, or pericardial effusion. •History of checkpoint inhibitor therapy. •The presence of a clinically detectable second primary malignancy, or history of other malignancies within 5 years. • A history of liver disease including, but not limited to, HBV infection or HBV DNA positive (≥1×10^4^/mL), HCV infection or HCV DNA positive (≥1×10^3^/mL), and liver cirrhosis. •Pregnant or lactating women or women who may be pregnant have a positive pregnancy test before the first medication, or the female participants themselves and their partners who were unwilling to implement strict contraception during the study period. •The investigator considers that the subject is not suitable to participate in this clinical study due to any clinical or laboratory abnormalities or compliance problems. • Serious mental abnormalities. • The diameter of brain metastasis is greater than 3 cm or the total volume is greater than 30 cc. • Clinical or radiological evidence of spinal cord compression, or tumors within 3 mm of the spinal cord on MRI.

### Screening

Patients will undergo a thorough screening process within 2 weeks prior to treatment in order to assess their tolerance. During this period, comprehensive information on potentially eligible patients will be diligently collected and recorded. The screening process will include several important steps such as obtaining written informed consent, collecting demographic information and medical history, conducting a physical examination, evaluating the ECOG PS score and vital signs, performing clinical testing (chemistry, hematology, and coagulation), assessing liver and kidney function, and conducting cardiac analyses. Tumor information will be obtained through imaging evaluations using computed tomography (CT), magnetic resonance imaging (MRI), or positron emission tomography/CT. Finally, the patient’s eligibility will be determined by reviewing the inclusion and exclusion criteria, leading to a final judgment.

### Interventions

#### Locally recurrent site radiotherapy

RT will be delivered using a linear accelerator that utilized 6-MV photons. Each patient will undergo a planning CT scan in the treatment position, and IMRT planning is employed based on the imaging from the planning CT. To determine the gross tumor volume (GTV), a combination of physical examination, CT, MRI, and/or PET-CT findings are taken into account.

For pelvic radiation-naive patients, the clinical target volume (CTV) comprises the GTV, internal iliac, pre-sacral, and peri-rectal nodal regions, external iliac nodal region (if positive external iliac lymph nodes are present), and inguinal nodal region (if positive inguinal nodes are present). In cases where available, the RTOG anorectal contouring atlas is followed to generate the CTV. A symmetrical margin of 5 mm is applied around the CTV to generate the planning target volume (PTV). Additionally, a PTVBOOST is generated by expanding the GTV symmetrically by 1 cm. The PTV receives a dose of 25 Gy in 5 treatment fractions, with a concomitant PTVBOOST dose of 30–40 Gy in 5 fractions based on organ-at-risk constraints.

For patients with a history of previous pelvic radiation, the PTV is generated with a symmetrical margin of 10 mm around the GTV. The RT dose for the PTV is 15–30 Gy in 5 treatment fractions, considering organ-at-risk constraints.

Organ-at-risk constraint calculation.

Five-fraction constraints (no previous radiotherapy):

Small bowel V35 ≤ 0.5 mL and V25≤ 1 0 mL, Large bowel/rectum V32 ≤ 0.5 mL, Bladder V38 ≤ 0.5 mL, Femoral head V30 ≤ 10 mL, Lumbosacral plexus V32 ≤ 0.5 mL and V30 ≤ 5 mL.

Five-fraction constraints (previous pelvic radiotherapy history):

Previous radiation therapy plan and dose volume histogram will be reviewed for relevant dose metrics. OARs delineated include small and large bowel, bladder, femoral head, and lumbosacral plexus.


$$\text{Re-irradiation constraint}=EQD2_{1}–(EQD2_{2}\times (1-TRF))$$

$$EQD2_{1}=D1\left(d1+\;\frac{\alpha }{\beta }\right)/\left(2+\;\frac{\alpha }{\beta }\right)$$

$$EQD2_{2}=D2\left(d2+\;\frac{\alpha }{\beta }\right)/\left(2+\;\frac{\alpha }{\beta }\right)$$

TRF= 0.15× years post-radiotherapy

where D1 is the relevant national UK SABR constraints (d1 per fraction constraint), D2 is the previous radiotherapy dose (d2 per fraction previous dose), and TRF is the tissue recovery factor (40, 41).

#### Oligometastases radiotherapy

For patients with oligometastases, defined as distant metastatic lesions being no more than 5 and involving no more than 3 organ sites, stereotactic ablative radiotherapy (SABR) will be performed on all metastasis sites. The GTV for each lesion will be defined as the visible tumor on CT and/or MRI imaging ± PET, and no additional margin will be added for microscopic disease spread (i.e., clinical target volume [CTV] = GTV). A PTV margin of 3–5 mm will be added depending on the site of disease and immobilization: 3-mm margins should be used for spinal stereotactic treatments and brain tumors, and 5 mm for other sites.

Each lesion will be treated with 5 fractions and all doses will be prescribed to the periphery of the PTV. Five-fraction regimens based on organ-at-risk constraints will be delivered daily, with a dose range of 35–50 Gy/5 Fx. SABR will be delivered between chemoimmunotherapy cycles, and NO systemic therapy agents are allowed during the period commencing 2 weeks prior to radiation and lasting until 1 week after the last fraction.

##### Organ at risk doses

OAR doses, as shown in **Tables 2**, **3**, must not exceed the specified limits. If achieving the desired PTV coverage would result in exceeding the OAR doses, priority should be given to compromising the PTV coverage. It is important to contour all OARs within 5 cm of the PTV (42).

**Table 2:** Dose constraints for serial structures.

Structure	Volume	5 Fraction
**Optic pathway**	D0.03cc	25
	D0.2cc	23
**Cochlea**	D0.03cc	22
**Brainstem**	D0.03cc	31
	D0.5cc	23
**Spinal Cord**	D0.03cc	28
	D0.35cc	22
**Cauda equina or sacral plexus**	D0.03cc	31.5
	D5cc	30
**Esophagus**	D0.03cc	35
	D5cc	19.5
**Brachial plexus**	D0.03cc	32.5
	D3cc	27
**Heart**	D0.03cc	38
	D15cc	32
**Great vessels**	D0.03cc	53
	D10cc	47
**Trachea and large bronchi** (mainstem, bronchus intermedius)	D0.03cc	40
	D4cc	—
	D5cc	32
**Chest wall or rib**	D0.03cc	57
	D5cc	45
**Skin**	D0.03cc	38.5
	D10cc	36.5
**Stomach**	D0.03cc	35
	D10cc	26.5
**Bile duct**	D0.03cc	41
**Duodenum**	D0.03cc	26
	D5cc	18.5
	D10cc	14.5
**Jejunum or ileum**	D0.03cc	32
	D30cc	20
**Colon**	D0.03cc	40
	D20cc	28.5
**Rectum**	D0.03cc	55
	D3.5cc	50
	D20cc	32.5
**Ureter**	D0.03cc	45
**Bladder**	D0.03cc	38
	D15cc	20
**Penile bulb**	D3cc	30
**Femoral heads**	D10cc	30

D0.03cc = maximum dose in Gy allowable to the hottest 0.03 cc; other D values are used in the same way.

**Table 3:** Dose constraints for parallel structures.

Structure	Volume	5 Fraction
**Lung (combined right and left, subtract GTVs)**	1,500 cc	12.5
**Liver**	700 cc	21
**Kidney cortex (combined left and right)**	200 cc	18

For example, for lung, meaning that there must be 1,500 cc of lung receiving 12.5 Gy or less.

#### Chemotherapy

Patients (cohort A) will receive CAPOX, FOLFIRI, or mFOLFOX6 chemotherapy based on previous adverse reactions to chemotherapy agents and at the discretion of the oncologist.

Patients (cohort B) will receive CAPOX, FOLFIRI, mFOLFOX6, mXELIRI, irinotecan and raltitrexed, or oxaliplatin and raltitrexed chemotherapy based on the first-line chemotherapy and previous adverse reactions to chemotherapy agents and at the discretion of the oncologist.

Treatment details will be as follows:

CAPOX: Capecitabine: 1,000 mg/m^2^ twice daily d1–14 q3w, Oxaliplatin: 130 mg/m^2^ d1 q3w.
FOLFIRI: irinotecan 180 mg/m^2^ on day 1 intravenously, folinic acid 400 mg/m^2^ on day 1, 5FU 400 mg/m^2^ intravenously on day 1, and 5 FU 2,400 mg/m^2^ continuous intravenous infusion on days 1 and 2.
mFOLFOX6: oxaliplatin 85 mg/m^2^ in 2 h on day 1 intravenously, folinic acid 400 mg/m^2^ on day 1, 5FU 400 mg/m^2^ intravenously on day 1, and 5FU 2,400 mg/m^2^ continuous intravenous infusion on days 1 and 2.
mXELIRI: irinotecan 200 mg/m^2^ intravenously on day 1 plus oral capecitabine 800 mg/m^2^ twice daily on days 1–14, repeated every 21 days.
Irinotecan and raltitrexed: irinotecan (200 mg/m^2^) and raltitrexed (3 mg/m^2^) were given intravenously on day 1. Cycles were repeated every 3 weeks.
Oxaliplatin and raltitrexed: oxaliplatin (130 mg/m^2^) and raltitrexed (3 mg/m^2^) were given intravenously on day 1. Cycles were repeated every 3 weeks.

After completing the (re)irradiation and 18 weeks of chemoimmunotherapy, the evaluation of patients will be discussed during the MDT meeting at our center. The purpose of this discussion is to determine whether the patients are suitable candidates for surgical resection. For patients who are not eligible for resection, long-term treatment will be continued. In cases where there is progressive response to the treatment, an exit strategy will be recommended. It is important to note that follow-up will be implemented specifically for patients who achieve a complete response (CR).

### Follow-up

Patients will undergo tumor assessment at baseline, with subsequent assessments scheduled every 9 weeks (± 7 days) for the first 12 months following treatment initiation. After this initial period, follow-up appointments will be scheduled every 3 months for a minimum of 2 years. Subsequently, follow-ups will be conducted every 6 months from the third to the fifth year. Finally, patients will have follow-up examinations once every 12 months for the rest of their lives. These follow-up examinations will encompass pelvic MRI/CT, abdominal MRI/CT, chest CT, and may include FDG-PET imaging as deemed necessary. Additionally, laboratory analysis of tumor markers in the blood will be performed.

### Outcome measures

The primary endpoint of the study is the local objective response rate, which is defined as the proportion of patients demonstrating a confirmed complete or partial response in the pelvic region according to RECIST 1.1 criteria and as assessed by the investigator. Secondary endpoints include the extrapelvic objective response rate, which measures the proportion of patients with confirmed complete or partial responses outside the pelvic region based on RECIST 1.1 criteria. Another secondary endpoint is the R0 resection rate, which determines the proportion of patients who achieve an R0 resection of pelvic recurrent tumors following therapy. In addition, the duration of response is evaluated as the time elapsed from the first documented pelvic objective response to either pelvic or extrapelvic disease progression in patients with confirmed response. Progression-free survival measures the time from the start of treatment until disease progression, with censoring at the last follow-up or death. Overall survival is defined as the time from the start of treatment until death from any cause, with censoring at the last follow-up. Finally, the safety and tolerability of the treatment are also assessed.

### Safety assessment

Safety evaluation during the TORCH-R trial will involve observation and documentation of adverse events (AEs) and serious AEs of any grade according to NCI-CTCAE 5.0. The assessment will incorporate various elements, including observations during treatment, laboratory analyses, electrocardiography, physical examinations, and ECOG PS scores. It will be the responsibility of investigators to carefully measure and document any AEs that occur, as well as determining the causal relationship between the observed AEs and the study drugs being administered. By conducting thorough safety evaluations, we aim to ensure the wellbeing of trial participants and identify any potential risks associated with the treatment regimen. This information will contribute to a comprehensive understanding of the safety profile of combining hypofractionated radiotherapy, chemotherapy, and toripalimab for LRRC.

### Statistical analysis

In this study, we will utilize SPSS 22.0 software for statistical analysis. For quantitative data that satisfy the requirements of normal distribution, we will express it as mean ± standard deviation. In cases where the data do not meet the normal distribution requirements, we will use median as a measure. Qualitative data will be presented as percentages (%), and a confidence level of 95% will be employed to calculate confidential intervals. To estimate the 95% confidence intervals of the objective response rate and R0 resection rate, we will utilize the Clopper–Pearson method. The median duration of response, progression-free survival, and overall survival will be calculated using the Kaplan–Meier method. To estimate their respective 95% confidence intervals, we will employ the Brookmeyer–Crowley method. All statistical tests conducted will be two-sided, and a significance level of *p*< 0.05 will indicate that the observed differences were statistically significant.

### Sample size calculation

In cohort A, we have a reference local ORR of 45% (P0) and assume that our treatment group can achieve an ORR of 65% (P1). We will use a two-sided test with a significance level of α = 0.05. The null hypothesis (H0) is that the local ORR of our treatment group is not better than the reference (P1 − P0 ≤ 0), while the alternative hypothesis (H1) is that the local ORR of our treatment group can be increased by 20% compared to the reference (P1 − P0 = 20%). To achieve a test efficiency of 80%, 48 patients need to be enrolled. Assuming a 10% dropout rate, a total of 53 patients are needed.

In cohort B, we have a reference local ORR of 20% (P0) and assume that our treatment group can achieve an ORR of 40% (P1). We will also use a two-sided test with a significance level of α = 0.05. The null hypothesis (H0) is that the local ORR of our treatment group is not better than the reference (P1 − P0 ≤ 0), while the alternative hypothesis (H1) is that the local ORR of our treatment group can be increased by 20% compared to the reference (P1 − P0 = 20%). To achieve a test efficiency of 80%, 36 patients need to be enrolled. Assuming a 10% dropout rate, a total of 40 patients are needed.

## Discussion

The optimal treatment for recurrent rectal cancer has not been established, although chemo(re)radiotherapy has been recommended before the widespread use of immunotherapy (43). With the success of immunotherapy in MSI-H CRC (44–46), more studies have explored the potential benefits of immunotherapy in MSS LARC. The TORCH study has provided some experience in combining neoadjuvant SCRT and immunotherapy for LARC. This combination therapy has shown potential to improve the rate of complete response and is well tolerated by patients (35, 36). Based on this background, the TORCH-R study aims to evaluate the safety and efficacy of immunotherapy plus chemo(re)radiotherapy for LRRC. The study aims to further improve the local response rate of pelvic recurrence tumor and overall prognosis for patients with LRRC.

Recent data have shown promising results for the synergy of immunotherapy with CRT in controlling or eradicating rectal cancer. Although these clinical trials have small sample sizes and are in phase II, their preliminary findings are encouraging. For example, the Japanese VOLTAGE-A study performed LCRT followed by five cycles of nivolumab and found that 30% reached pCR and 8% reached near-pCR, while one patient achieved cCR and underwent the W&W strategy in MSS patients (26). The NRG-GI002 trial used eight cycles of FOLFOX followed by LCRT (concurrent with capecitabine and pembrolizumab) and showed that the pCR+cCR rates were 44% (31). The Averectal trial used SCRT followed by six cycles of mFOLFOX6 and avelumab, resulting in a 37.5% pCR rate and 30% of patients achieving near-pCR (TRG 1) (34). Another Chinese study used SCRT followed by two cycles of XELOX and camrelizumab, with a 46% pCR rate in MSS patients (36). While the VOLTAGE-A study had grade 3–4 immune-related toxicities in 7.7% of patients, no grade 3–4 immune-related adverse effects were observed in the two SCRT studies. These studies suggest that combining immunotherapy with preoperative CRT can result in good tumor downstaging and tolerability in LRRC.

It is crucial to consider the fraction of radiation when realizing the full potential of immunotherapy-radiotherapy regimens. The evidence presented suggests that SCRT combined with immunotherapy may have superior pCR rates and lower toxicity compared to LCRT. Preclinical studies investigating different dose and fraction regimens have concluded that larger doses per fraction are associated with optimal immune stimulation effect (47, 48). Hypofractionated radiation doses induce DNA damage more effectively than lower doses, leading to greater formation of micronuclei and cytoplasmic leakage of DNA, resulting in the production of more IFN-I in the radiated tumor cell (49). Additionally, hypofractionated radiotherapy can inhibit the recruitment of myeloid-derived suppressor cells (MDSCs) to tumors and achieve better tumor growth inhibition than conventional fractionation in mice (38). Therefore, a combination model of hypofractionated radiotherapy (25–35 Gy/5 Fx for radiation-naive patients and 15–30 Gy/5 Fx for patients with pelvic radiation history), chemotherapy, and immunotherapy was adopted to investigate whether adding immunotherapy to the CRT approach could lead to an improved response rate and prognosis in LRRC patients.

It has been observed that approximately 50% of rectal cancer recurrences have synchronous metastatic disease at the time of diagnosis, and 30%–42% develop metachronous metastatic disease after resection (50, 51). In patients with metastatic disease, high-dose hypofractionated radiation, such as SABR, has been widely used for curative or palliative purposes. SABR not only effectively destroys tumor cells directly, but it may also stimulate anti-tumor vaccine *in situ* to prime the immune system (52). However, only a few studies have described the occurrence of the abscopal effect, where tumor-specific T cells target cancer cells at metastatic sites outside the radiation fields (52, 53). This rare occurrence suggests that radiation in one tumor lesion is insufficient to overcome pre-existing suppression or tolerance of anti-tumor immune responses. In order to maximize the immune stimulation induced by radiation and enhance the efficacy of immunotherapy, all metastasis sites will receive SABR in this study. This approach aims to not only directly destroy tumor cells but also trigger a systemic immune response against the cancer. By treating the metastatic sites with SABR, we hope to achieve better control of the disease and potentially improve patient outcomes.

Indeed, there is a need for standardized treatment strategies and improved outcomes for LRRC. Based on our preliminary study results in LARC, it suggests that combining preoperative immunotherapy with traditional treatment methods like chemoradiotherapy (CRT) can result in good efficacy. To address this, the TORCH-R clinical trial has been initiated. Its objective is to investigate whether adding immunotherapy to CRT can lead to improved overall response rate (ORR), R0 resection rate (complete tumor removal), better tolerance to treatment, and, ultimately, a better prognosis for LRRC patients. The aim is to evaluate the potential benefits of integrating immunotherapy into the current treatment approach for LRRC. This trial represents an important step forward in exploring novel therapeutic strategies to enhance outcomes in LRRC patients.

## Data availability statement

The original contributions presented in the study are included in the article/supplementary material. Further inquiries can be directed to the corresponding author.

## Ethics statement

The studies involving humans were approved by Ethics Committee of Fudan University Shanghai Cancer Center. The studies were conducted in accordance with the local legislation and institutional requirements. The participants provided their written informed consent to participate in this study.

## Author contributions

JW: Writing – original draft, Writing – review & editing. RW: Writing – original draft, Writing – review & editing. MF: Writing – original draft, Writing – review & editing. LS: Writing – original draft, Writing – review & editing. HZ: Writing – review & editing. YW: Investigation, Resources, Writing – review & editing. YQW: Investigation, Resources, Writing – review & editing. SZ: Investigation, Resources, Writing – review & editing. YC: Investigation, Resources, Writing – review & editing. FX: Investigation, Resources, Writing – review & editing. ZZ: Writing – review & editing.
